# Multichannel High Resolution Wide Swath SAR Imaging for Hypersonic Air Vehicle with Curved Trajectory

**DOI:** 10.3390/s18020411

**Published:** 2018-01-31

**Authors:** Rui Zhou, Jinping Sun, Yuxin Hu, Yaolong Qi

**Affiliations:** 1 Electronics & Information Engineering, Beihang University, Beijing 100191, China; zhourui2088@buaa.edu.cn (R.Z.); longgniy@163.com (Y.Q.); 2 Key Laboratory of Technology in Geospatial Information Processing and Application System, Institute of Electronics, Chinese Academy of Sciences, Beijing 100191, China; yxhu@mail.ie.ac.cn

**Keywords:** hypersonic air vehicle, azimuth multichannel sampling, DPCA, high resolution wide swath (HRWS), ETF imaging algorithm, near space, curved trajectory

## Abstract

Synthetic aperture radar (SAR) equipped on the hypersonic air vehicle in near space has many advantages over the conventional airborne SAR. However, its high-speed maneuvering characteristics with curved trajectory result in serious range migration, and exacerbate the contradiction between the high resolution and wide swath. To solve this problem, this paper establishes the imaging geometrical model matched with the flight trajectory of the hypersonic platform and the multichannel azimuth sampling model based on the displaced phase center antenna (DPCA) technology. Furthermore, based on the multichannel signal reconstruction theory, a more efficient spectrum reconstruction model using discrete Fourier transform is proposed to obtain the azimuth uniform sampling data. Due to the high complexity of the slant range model, it is difficult to deduce the processing algorithm for SAR imaging. Thus, an approximate range model is derived based on the minimax criterion, and the optimal second-order approximate coefficients of cosine function are obtained using the two-population coevolutionary algorithm. On this basis, aiming at the problem that the traditional Omega-K algorithm cannot compensate the residual phase with the difficulty of Stolt mapping along the range frequency axis, this paper proposes an Exact Transfer Function (ETF) algorithm for SAR imaging, and presents a method of range division to achieve wide swath imaging. Simulation results verify the effectiveness of the ETF imaging algorithm.

## 1. Introduction

With the development of synthetic aperture radar (SAR) technology, the SAR carrier has been developed from the initial airborne and spaceborne platforms to the unmanned aerial vehicle, missile and vehicle-mounted platforms [1,2]. The near space, as a new space outside the traditional active area of vehicles, has attracted wide attention for its unique advantages in recent years, and the relevant exploring research has become a new topic in SAR imaging [3,4].

Compared with the conventional airborne platform, the hypersonic platform mainly works in the typical altitude of 20–100 km, which helps to achieve a wider range of observation. Meanwhile, the integrated design of this platform is considered to improve the stealth performance, and the cruising speed is usually more than 5 Mach to achieve the fast response. Therefore, the detection, tracking and interception probability on this platform would be relatively low for the hostile defense system [5,6]. In addition, the flight trajectory of this platform is not constrained by the special orbits compared with the spaceborne platform. Thus, the flexible maneuvering characteristics are beneficial to reducing the revisit time on the coverage of the focused area [7].

The spatial geometric variation between the radar platform and the targets located in the imaging scene determines the Doppler history of SAR echo signals. Therefore, it is a key point to construct an appropriate signal model relying on the motion trajectory and the imaging mode of the SAR platform. For airborne SAR, when the turning slope angle and the flight path angle of the vehicle are constants, the turning radius is determined by its instantaneous velocity [8]. In general, the SAR imaging geometry model of the conventional airborne platform is established based on the linear flight trajectory. In contrast, to avoid the tracking and interception of the hostile defense system, the flight path of the hypersonic platform undertaking the reconnaissance task is planned as a curved trajectory. Moreover, the turning radius of this platform could be hundreds of kilometers because of its high instantaneous velocity. Thus, the motion trajectory is characterized as an approximate arc shape, which forms an arc band-shaped coverage. Affected by the curved trajectory of this platform, the range migration becomes more obvious. At this point, when the SAR imaging is processed with the traditional hyperbolic range model, the imaging quality of targets would be seriously degraded, or even cannot satisfy the performance requirements of the SAR system. For this problem, an accurate range model matched with the curved trajectory of the hypersonic platform is presented in this paper.

In addition, under the constraint of minimum antenna area [9], the contradiction between the high resolution and wide swath (HRWS) becomes more serious for the hypersonic platform SAR. With a large increase of this platform velocity, a higher Doppler bandwidth is required to achieve the specific azimuth resolution. Accordingly, the pulse repletion frequency (PRF) should be higher to avoid azimuth ambiguity. However, the high PRF will limit the swath range; otherwise, it will make range ambiguity worse. Therefore, for the conventional single channel SAR system, the PRF selection is compromised between HRWS requirements [10]. To alleviate this contradiction, Currie [11] proposed the multichannel receiving displaced phase center antenna (DPCA) technique, based on which the Doppler ambiguity resulted from a low PRF can be separated by increasing the spatial sampling degrees of freedom. On this basis, Krieger [12] discussed the advantages of applying the azimuth multichannel transceiver technology in detail, and Cerutti-maori et al. [13,14,15] verified the effectiveness of HRWS applications with the simulation results of the traditional spaceborne and airborne platform. Inspired by this, the hypersonic platform SAR adopts the displaced phase center antenna multichannel (DPCAM) technology to meet the HRWS imaging requirement in this paper.

Although the DPCAM technique can effectively widen the range swath for hypersonic platform, there are strict restrictions on SAR system parameters such as the velocity of the platform, PRF and the phase center spacing of sub-apertures. Any deviation of parameters will lead to the non-uniform sampling in azimuth [16,17]. However, due to the curved trajectory of the hypersonic platform SAR, each sub-aperture is linearly configured along the tangential direction of the flight trajectory, thus the azimuth data is difficult to meet the uniform sampling condition. Moreover, the PRF selection should be diverse since it is required to satisfy different beam position design. In addition, the velocity of this platform cannot hold as a constant. All of these factors would induce a non-uniform sampling in azimuth. Thus, based on the multichannel signal reconstruction theory [18], Quan et al. [19,20] proposed the spectrum reconstruction methods for SAR azimuth non-uniform sampling to alleviate the limitation of PRF selection. In this paper, we present a more efficient spectrum reconstruction model using the discrete Fourier transform and multiplication of complex data, which can pre-eliminate the effects of azimuth non-uniform sampling.

According to the curved trajectory of the hypersonic platform SAR, the range-azimuth coupling will show a strong space-varying property, which further increases the difficulty for SAR imaging. Similar to the imaging geometry of the hypersonic platform, Lee [21] studied the truck-mounted arc-scanning SAR, where the measured results with different imaging modes mainly focused on the monitoring application of the terrain deformation. Moreover, to improve the azimuth resolution of the rotating synthetic aperture radar (ROSAR) with circular trajectory, Wang et al. [22] derived the approximate linear sampling model based on resampling and rotational transformation processing, and the extended Omega-K algorithm is used to obtain the ideal imaging results. In addition, since the second-order Taylor series expansion of the slant range will limit the effective synthetic aperture length and the range of reconstruction angle for ROSAR, Kou et al. [23,24] put forward the modified Omega-K algorithm to improve the imaging results by keeping higher order terms of Taylor series expansion. Although the traditional Omega-K algorithm adopting the hyperbolic range model can accurately correct the range cell migration, and obtain a well-focused SAR image for hypersonic platform SAR, this algorithm can only accurately focus the targets located at the reference range, and the residual phase at the non-reference range cannot be compensated by the Stolt interpolation operation. Meanwhile, it is difficult to deduce the imaging algorithm for hypersonic platform SAR due to the complexity of the slant range model. In this paper, an equivalent range model based on the minimax criterion is proposed, where the optimal second-order fitting coefficients of cosine function are derived using the two-population coevolutionary algorithm. On this basis, an Exact Transfer Function (ETF) imaging algorithm is then designed for hypersonic platform SAR with curved trajectory.

## 2. Signal Model

In this section, according to the curved trajectory of the hypersonic platform, the SAR imaging geometry model and the multichannel sampling model in azimuth are established, respectively. Then, the equivalent space sampling of each sub-aperture is analyzed and the accurate slant range model under curved trajectory is introduced.

### 2.1. Imaging Geometry Model

**Figure 1:**
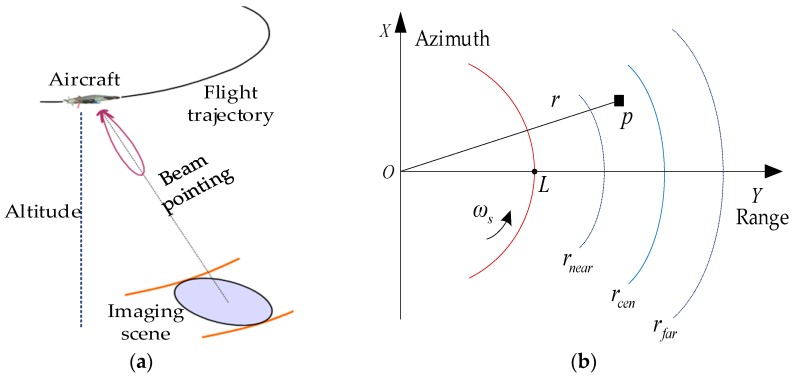
SAR imaging model for hypersonic platform: (**a**) geometric diagram; (**b**) plane diagram.

As shown in Figure 1, the hypersonic platform SAR moves along an approximate arc trajectory. Accordingly, an arc band-shaped observation scene can be illuminated by fixing the antenna beam pointing. Here, L represents the turning radius of the platform, $\omega_{s}$ denotes the angular velocity, r stands for arbitrary position of target p in the imaging scene, and $r_{cen}$ is the center of the scene.

### 2.2. Azimuth Transceiver Model

The hypersonic platform SAR adopts the DPCAM technique, that is, multiple sub-apertures are configured in the along-track dimension, and each sub-aperture has the same beamwidth and illuminates the same observation scene. For the single transmitter and multiple receivers sampling mode of SAR system, each receiving sub-aperture can obtain the independent azimuth echo signal such that PRF can be reduced by adjusting the phase center spacing of the adjacent sub-apertures.

**Figure 2:**
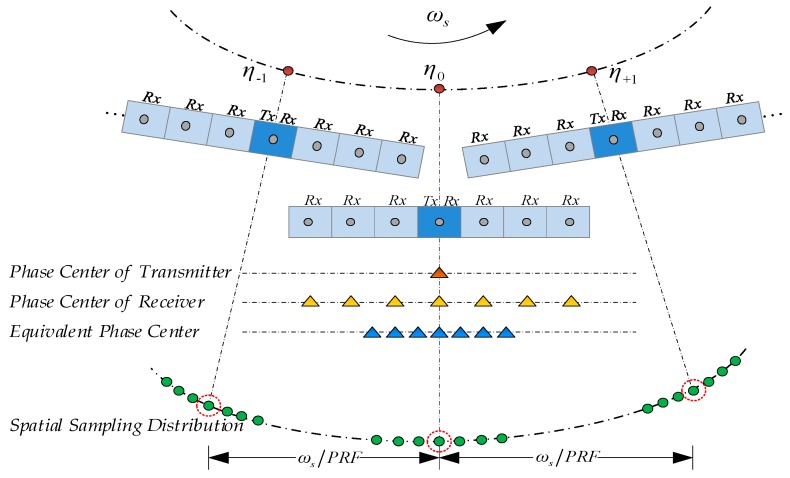
Azimuth multichannel sampling for hypersonic platform SAR.

To meet a certain range of imaging bandwidth, as an example shown in Figure 2, azimuth multichannel sampling for hypersonic platform SAR adopts the mode of single transmitter and multiple receivers, that is, the middle sub-aperture transmits the signal and all of the sub-apertures receive the echo signal. For conventional airborne SAR, the array antenna configuration is adapted with the flight trajectory of the platform. However, due to the curved trajectory of the hypersonic platform, only the instantaneous position of the transceiver sub-aperture coincides with the current flight trajectory, while all other receiving sub-apertures deviate from the flight trajectory. Since the instantaneous turning radius of this platform is much larger than the phase center spacing of sub-apertures, the equivalent sampling position of each sub-aperture can be approximately processed along the curved trajectory. In this way, it is helpful that the azimuth receiving model could be simplified with some minor sampling position error.

### 2.3. Equivalent Range Model

Since the SAR slant range defined from the antenna phase center to the targets largely determines the focusing accuracy of the targets [25], thus the accurate slant range form was derived based on the SAR imaging geometry model of the hypersonic platform.

For DPCAM SAR of the hypersonic platform, each sub-aperture is arranged linearly in the along-track dimensional, thus the qth receiving sub-aperture deviates from the center of the transmitting sub-aperture can be expressed by

(1) $$△x_{q}=\left(\frac{Q+1}{2}-q\right)d,\;q=1,2,\cdots ,Q,$$

where d is the spacing of the adjacent phase center of receiving sub-apertures, and Q is the number of receiving sub-apertures, which is an odd number.

Refer to Figure 2, the instantaneous slant range from the phase center of the transceiver sub-aperture to the targets can be expressed by

(2) $$R_{a}(\eta )=\sqrt{L^{2}+r^{2}-2Lr\cos\left(\omega_{s}\eta \right)+h^{2}}.$$

Furthermore, since the azimuth multichannel sampling of the hypersonic platform SAR adopts the mode of single transmitter and multiple receivers, based on which the slant range from the phase center of the transmitter to the targets can be substituted by the instantaneous slant range of the transceiver sub-aperture. Then, the accurate two-way slant range of this platform SAR can be given by

(3) $$R_{arc,q}\left(\eta \right)=R_{a}\left(\eta \right)+\sqrt{L^{2}+r^{2}-2Lr\cos\left(\omega_{s}\eta -△x_{q}/L\right)+h^{2}}.$$

## 3. Azimuth Spectrum Reconstruction

In view of the azimuth non-uniform sampling for hypersonic platform SAR, this section first analyzes the relative Doppler parameters, and then provides a high-efficient algorithm to reconstruct the azimuth echo signal, which can pre-eliminate the effects of the spectrum ambiguity on SAR imaging.

### 3.1. Doppler Parameters Analysis

As shown in Figure 1, the angular velocity of the hypersonic platform can be defined as $\omega_{s}=V_{s}/L$, and the corresponding ground linear velocity of the illuminated target is defined as $V_{G}=\omega_{s}r$. Then, the SAR Doppler rate can be derived based on the second-order Taylor series expansion of Equation (2), i.e.,

(4) $$K_{a}=-\frac{2\omega_{s}^{2}Lr}{\lambda R}=-\frac{2V_{s}V_{G}}{\lambda R},$$

where R is the closest slant range of targets. Due to $V_{G}>V_{s}$, the Doppler rate of side-looking SAR for hypersonic platform will be larger compared with the traditional airborne SAR. 

In addition, the synthetic aperture time can be deduced according to the imaging geometry model of the hypersonic platform, which is given by

(5) $$T_{syn}=\frac{R\theta_{a}}{V_{G}}=\frac{R\theta_{a}}{\omega_{s}r},$$

where $\theta_{a}$ denotes the beamwidth in azimuth. Similarly, due to r>L, SAR synthetic aperture time for hypersonic platform will be shorter.

Based on Equations (4) and (5), the Doppler bandwidth can be further obtained by

(6) $$B_{a}=K_{a}T_{syn}=\frac{2V_{s}}{D_{a}},$$

where $D_{a}$ is the length of sub-aperture. From Equation (6), it can be noted that the SAR Doppler bandwidth for hypersonic platform holds as a constant compared with the conventional airborne SAR. Furthermore, the SAR theoretical minimum azimuth resolution can be given as

(7) $$\rho_{a}=\frac{V_{G}}{B_{a}}=\frac{\lambda r}{2L\theta_{a}}.$$

### 3.2. Azimuth Spectrum Reconstruction

As shown in Figure 2, to achieve HRWS imaging, the hypersonic platform SAR adopting the DPCAM technique has the access to permit a low PRF in azimuth sampling. Based on which multiple sub-apertures are linearly configured in the along-track dimensional and each of them deviates from the transceiver, then azimuth sampling signal of the multiple sub-apertures can be obtained in one pulse repetition time (PRT). However, since the DPCAM equivalent azimuth echo is a non-uniform sampling sequence, which cannot be directly processed as an input signal for SAR imaging. Thus, in this section, the non-uniform sampling signal is pre-processed using a more efficient spectrum reconstruction method, and then the azimuth uniform signal of the equivalent single channel SAR system with low PRF can be obtained. 

Generally, based on the multichannel signal reconstruction theory [18], the multi-filter banks reconstruction method for SAR non-uniform sampling mainly includes the linear filter banks and reconstruction filter banks. Specially, the azimuth baseband signal with a limited bandwidth $B_{a}$ is passed through the Q band-limited filter banks, and the filtered signal is further sampled at the under-sampling rate of $B_{a}/Q$. Then, the multichannel reconstruction filter banks should be designed to restore the azimuth uniform signal. Based on the azimuth response of the single channel SAR system, Krieger et al. [20] derived the transfer function of the linear filter banks by introducing a constant phase shift and a time delay. Furthermore, the reconstruction filter banks are derived from the Q transfer functions. In this paper, we present a new reconstruction method with only fast Fourier transform (FFT) and multiplication of complex data, thus the high complexity of implementing the general interpolation operation can be effectively avoided.

When the DPCAM uniform sampling condition is not satisfied, although the azimuth signal is shown as a non-uniform sampling sequence, the adjacent Q sampling points are uniformly spaced in azimuth time. Therefore, we consider that the non-uniform azimuth signal is generated after sampling the filtered signal of Q channels uniformly. Here, the impulse response of the qth linear filter is recorded as $\left\{\delta (\eta -\Delta x_{q}/2L\omega_{s})\right\}$. Then, the transfer function of each pre-filter bank can be expressed as

(8) $$H_{q}\left(f_{\eta }\right)=\exp\left(-j2\pi \frac{△x_{q}}{2L\omega_{s}}f_{\eta }\right).$$

The reconstruction filter banks can be further derived based on the linear filter banks, and each reconstruction filter is composed of Q band-limited filter banks, based on which the frequency range of the azimuth baseband signal can be further divided. Here, consider an odd number of receiving channels. Then, the frequency of the qth sub-band can be obtained by

(9) $$E_{q}=\left(-\frac{Q\cdot PRF}{2}+\left(q-1\right)\cdot PRF,-\frac{Q\cdot PRF}{2}+q\cdot PRF\right).$$

Referring to Equation (8), the independent pre-filter bank of each receiving channel can be further expressed as matrix $H(f_{\eta })$, which is

(10) $$H\left(f_{\eta }\right)=\left[\begin{array}{ccc} H_{1}\left(f_{\eta }\right) & \cdots & H_{Q}\left(f_{\eta }\right)\\ H_{1}\left(f_{\eta }+PRF\right) & \cdots & H_{Q}\left(f_{\eta }+PRF\right)\\ \vdots & \ddots & \vdots \\ H_{1}\left(f_{\eta }+\left(Q-1\right)PRF\right) & \cdots & H_{Q}\left(f_{\eta }+\left(Q-1\right)PRF\right) \end{array}\right],$$

where $f_{\eta }\in E_{1}$, and the qth row corresponds to the pre-filter function of the $E_{q}$ frequency range, and the qth column corresponds to the pre-filter function of the qth receiving channel.

Then, the inverse transformation of Equation (10) is performed to derive the reconstruction filters, where the corresponding matrix expression is expressed as follows:

(11) $$G\left(f_{\eta }\right)=H^{-1}\left(f_{\eta }\right)=\left[\begin{array}{cccc} G_{11}\left(f_{\eta }\right) & G_{12}\left(f_{\eta }+PRF\right) & \cdots & G_{1Q}\left(f_{\eta }+\left(Q-1\right)PRF\right)\\ G_{21}\left(f_{\eta }\right) & G_{22}\left(f_{\eta }+PRF\right) & \cdots & G_{2Q}\left(f_{\eta }+\left(Q-1\right)PRF\right)\\ \vdots & & \ddots & \vdots \\ G_{Q1}\left(f_{\eta }\right) & G_{Q2}\left(f_{\eta }+PRF\right) & \cdots & G_{QQ}\left(f_{\eta }+\left(Q-1\right)PRF\right) \end{array}\right],$$

where the qth row corresponds to the reconstruction filter $G_{q}(f_{\eta })$ of the qth receiving channel, and the qth column corresponds to the reconstruction filter function of the $E_{q}$ frequency range. 

The SAR azimuth echo signal at any discrete time can be obtained from the impulse response of the multichannel pre-filter banks and the reconstruction filter banks. The reconstructed azimuth signal can be expressed as

(12) $${\tilde{h}}_{a}\left(\eta \right)=\underset{n}{\Sigma }\underset{q}{\Sigma }h_{a}\left(n/PRF-\Delta x_{q}/2L\omega_{s}\right)g_{q}\left(\eta -n/PRF\right),$$

where $g_{q}(\eta )$ denotes the impulse response of the qth reconstruction filter. To further obtain the equally spaced azimuth signal, that is, the sequence of which is expressed as $\left\{{\tilde{h}}_{a}(m/(Q\cdot PRF))\right\}$. Then, Equation (12) can be rewritten as

(13) $$\begin{array}{ll} {\tilde{h}}_{a}\left(\frac{m}{Q\cdot PRF}\right) & =\underset{n}{\Sigma }\underset{q}{\Sigma }h_{a}\left(\frac{n}{PRF}-\frac{\Delta x_{q}}{2L\omega_{s}}\right)g_{q}\left(\frac{m}{Q\cdot PRF}-\frac{n}{PRF}\right)\\ & =\underset{n}{\Sigma }\underset{q}{\Sigma }h_{a}\left(\frac{Q\cdot n}{Q\cdot PRF}-\frac{\Delta x_{q}}{2L\omega_{s}}\right)g_{q}\left(\frac{m}{Q\cdot PRF}-\frac{Q\cdot n}{Q\cdot PRF}\right) \end{array}.$$

Furthermore, variable Q·n in Equation (13) can be substituted by

(14) $$z_{q}(p)=\{\begin{array}{ll} h_{a}\left(\frac{p}{Q\cdot PRF}-\frac{\Delta x_{q}}{2L\omega_{s}}\right), & p=Q\cdot n,\\ 0, & p\neq Q\cdot n. \end{array}$$

From Equation (13) and Equation (14), the uniformly spaced azimuth sampling can be further expressed as a sequence of convolutions, which is given by

(15) $${\tilde{h}}_{a}\left(\frac{m}{Q\cdot PRF}\right)=\underset{q}{\Sigma }z_{q}\left(\frac{m}{Q\cdot PRF}\right)⊗g_{q}\left(\frac{m}{Q\cdot PRF}\right).$$

**Figure 3:**
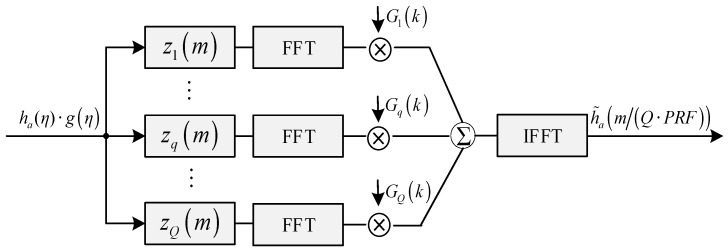
SAR spectrum reconstruction for azimuth multichannel non-uniform sampling.

Since the convolution calculation of discrete sequences can be realized by the FFT operation, thus the model of azimuth spectrum reconstruction can be further simplified, which is shown in Figure 3. Here, $\left\{G_{q}(k)\right\}$ is the discrete fourier transform of $\left\{g_{q}(m/(Q\cdot PRF))\right\}$.

## 4. HRWS Imaging Processing

The hypersonic platform SAR adopting the DPCAM technique can obtain the uniform azimuth echo signal after the spectrum reconstruction; then, the SAR imaging algorithm can be further designed to process the echo data. Due to the curved trajectory of this platform, when the traditional Omega-K algorithm is utilized, the precise focus of the targets located at the reference range will be achieved after the bulk compression processing. However, the residual phase of targets at the non-reference range is difficult to be compensated because the residual phase is not only related to the range frequency and the azimuth frequency, but the range variable r is also involved. Due to the fact that the Stolt mapping along the range axis cannot be implemented, the traditional Omega-K imaging algorithm cannot fully achieve the focus of the targets for hypersonic platform SAR with curved trajectory. To solve this problem, the ETF algorithm is designed in this section, and the SAR imaging for the wide swath is further considered by using the method of range division.

As given in Equation (2), the SAR slant range of the hypersonic platform is relatively complex, thus it is difficult to derive the two-dimensional spectrum of the SAR echo signal due to the existence of the cosine function. Meanwhile, it is not easy for the mutual coupled range-azimuth frequency to carry out the subsequent imaging processing. For this problem, the approximate processing of the SAR slant range is executed to reduce the complexity of the ETF imaging algorithm. Here, the derived hyperbolic range model using the second-order Tylor series expansion is beneficial for deducing the approximate spectrum of SAR echo signal due to its relatively simple expression. However, under the circumstance of the enhanced space-variant feature resulted from the curved flight trajectory, the ignored high-order terms will further increase the phase error in azimuth. Furthermore, the fourth-order or higher-order term of the Taylor series expansion is considered to approximate the slant range, then the focusing performance of the targets in azimuth can be effectively improved, but the computational complexity will be significantly increased. Therefore, from the point of the lower complexity and wider applicability, the cosine function existed in the SAR slant range form is directly processed using a second-order polynomial fitting. The equivalent range model without any series expansion is helpful to improve the azimuth phase precision. Furthermore, the difference of the approximate cosine function relative to the original cosine function is constructed as the object function, where the analytic form is a function of the azimuth angle and polynomial fitting coefficients. Then the optimal fitting coefficients can be obtained to minimize the maximum approximation error under different azimuth angle variables when the objective function is approximated. It is worth noting that the solution of the polynomial coefficients can be further transformed into a minimax optimization problem, thus the fitting coefficients are further solved by using the two-population coevolutionary algorithm. Then, the approximate processing of SAR slant range is completed.

Based on the approximate model of SAR slant range, we further design the ETF imaging algorithm. Here, the implementation of the imaging algorithm mainly includes the derivation of the two-dimensional spectrum, and the design of the exact transfer function and the compensation transfer function. Meanwhile, the ETF algorithm generally restricts the SAR imaging swath to avoid the defocus of targets near swath edge. Therefore, the wide swath will be divided into limited sub-swaths under the constraint of the ETF imaging algorithm. Then, the independent imaging processing on each sub-swath and the subsequent image mosaic operation are executed to obtain an integrate SAR image.

### 4.1. SAR Range Model

The second-order and fourth-order Taylor series expansion are executed to approximate the instantaneous range of Equation (2) respectively, then the hyperbolic range model and higher-order range model are given as

(16) $$R_{a1}(\eta )=R_{0}+\frac{Lr\omega_{s}^{2}\eta^{2}}{2R_{0}},$$

(17) $$R_{a2}(\eta )=R_{0}+\frac{Lr\omega_{s}^{2}\eta^{2}}{2R_{0}}-\frac{Lr\omega_{s}^{4}\eta^{4}}{24R_{0}}-\frac{L^{2}r^{2}\omega_{s}^{4}\eta^{4}}{8R_{0}^{3}},$$

where $R_{0}=\sqrt{{\left(r-L\right)}^{2}+h^{2}}$.

According to the SAR system parameters setting of the hypersonic platform, as shown in Table 1, the azimuth phase errors resulted from the approximate expansion of the slant range are calculated, respectively.

**Figure 4:**
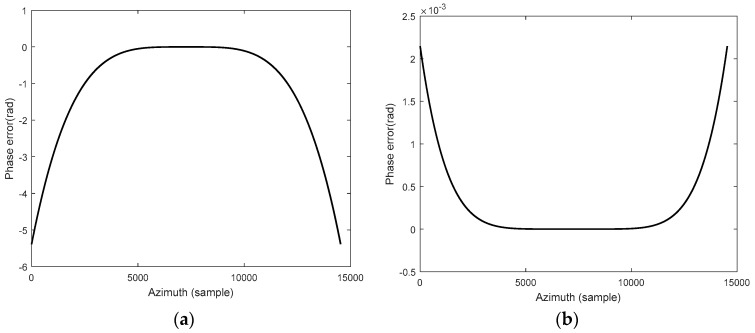
SAR azimuth phase errors introduced by approximate expansion of slant range: (**a**) Second-order Taylor series expansion; (**b**) fourth-order Taylor series expansion.

As shown in Figure 4a, the expression of the hyperbolic range model is relatively simple, thus it is helpful to execute the subsequent imaging processing and improve the calculation efficiency; however, the introduced azimuth phase error is more than π/4, which results in the degradation of the imaging quality. Moreover, when the fourth-order series expansion is used to approximate the instantaneous slant range, as shown in Figure 4b, the introduced azimuth phase error is less than π/4, thus the effects on subsequent imaging processing can be ignored. However, the high-order form of the SAR slant range is relatively complex, which results in high computational complexity for SAR imaging. Therefore, the optimal approximate processing of the cosine function is carried out further.

Based on the SAR imaging geometry model shown in Figure 1, the azimuth dimension can be redefined as an angular variation varying with the azimuth time, that is, $\theta (\eta )=\omega_{s}\eta$. Then, Equation (2) is rewritten as

(18) $$R_{a}(\theta )=\sqrt{L^{2}+r^{2}-2Lr\cos\theta +h^{2}}.$$

The cosine function in Equation (18) is further constructed as a second-order polynomial form, which is

(19) $$\cos\theta =\beta_{0}+\beta_{1}\theta^{2}.$$

Based on Equation (19), the solving of fitting coefficients $\beta_{0}$ and $\beta_{1}$ will be transformed into a minimax optimization problem, and its optimal solution is the minimum over feasible domain β of the maximum over feasible domain θ on the objective function Γ(β,θ). Mathematically, this minimax optimization problem is formally defined as

(20) $$\min_{\beta_{0},\beta_{1}}\;\max_{\theta }\;\Gamma \left(\beta ,\theta \right)=\min_{\beta_{0},\beta_{1}}\;\max_{\theta }\left|\cos\theta -\left(\beta_{0}+\beta_{1}\theta^{2}\right)\right|,$$

where the search interval of θ is set as $Y_{\theta }=[-\alpha_{theta}/2,\alpha_{theta}/2]$; here, $\alpha_{theta}$ is the azimuth beam width of the single channel SAR system. Moreover, the search interval of $\beta_{0}$ and $\beta_{1}$ are set as $X_{\beta }=[-5,5]$. 

Using an intelligent optimization algorithm for a certain population is the most intuitive way to solve the minimax problem above, that is, multiple independent sampling points of $\beta_{0}$ and $\beta_{1}$ are first randomly selected from population $X_{\beta }$, and the approximate solution of each sampling sequence can be solved as ${\tilde{\theta }}_{s}=\arg\;\max_{\theta \in Y_{\theta }}\Gamma ({\hat{\beta }}_{s},\theta )$. Then, function value $\Gamma ({\hat{\beta }}_{s},{\tilde{\theta }}_{s})$ corresponding to ${\hat{\beta }}_{s}$ is updated, and the approximate solution is solved as $\left({\tilde{\beta }}_{0},{\tilde{\beta }}_{1})={\hat{\beta }}_{s^{*}},s^{*}=\arg\;\min_{s}\Gamma ({\hat{\beta }}_{s},{\tilde{\theta }}_{s}\right)$. However, a global optimization algorithm solely used will result in high computational complexity to obtain ${\tilde{\theta }}_{s}$. Therefore, to further get the value of $\hat{\beta }$ more efficiently, this paper presents the two-population coevolutionary algorithm based on the meta-heuristic random searching algorithm [26,27].

**Figure 5:**
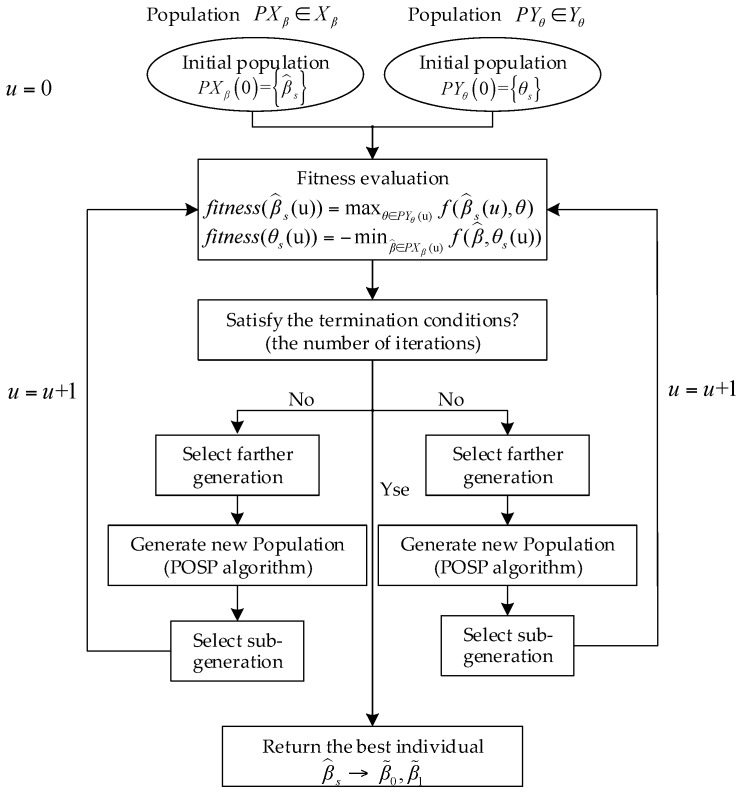
The flowchart of solving fitting coefficients using the two-population coevolutionary algorithm.

As shown in Figure 5, the independent individuals belonging to the global population are first sampled partly to form a new limited population, which is beneficial to reduce the complexity of the minimax optimization problem. Furthermore, the Particle Swarm Optimization (PSO) algorithm is utilized to generate a new population due to its advantages such as the less parameters setting, fast convergence and simple operation [28]. Each particle in the population contains the values of the current position, the best historical position and the velocity. In the search space, the single particle constantly moves according to the best historical position of itself and others particles; then, the optimal population belonging to the next generation is selected through the fitness evaluation [29,30]. After a specified number of iterations, the estimates of $\beta_{0}$ and $\beta_{1}$ are further derived from the returned optimal individual.

**Figure 6:**
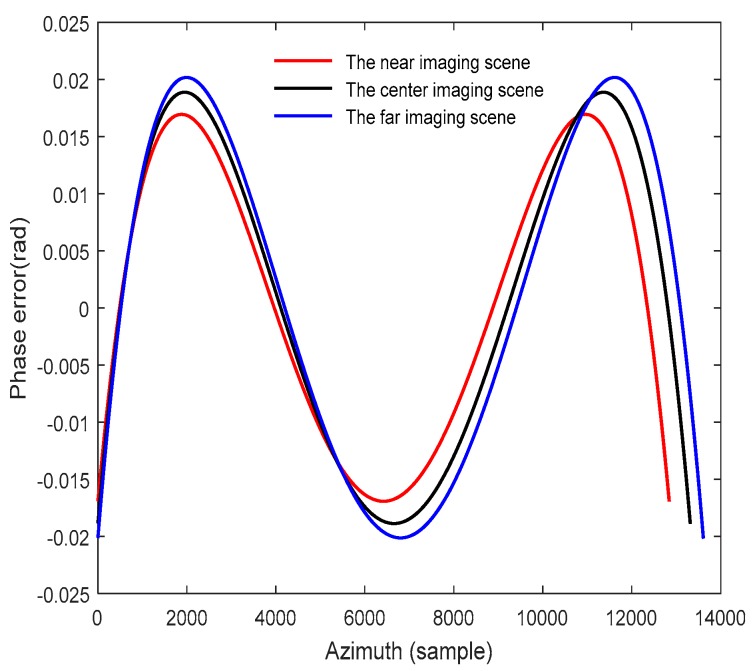
The phase errors introduced by the approximate polynomial fitting of the SAR slant range.

Based on the analysis above, the approximate model of the SAR slant range can be obtained from the derived fitting coefficients using the two-population coevolutionary algorithm. Then, the azimuth phase errors introduced by the approximate slant range model are calculated for different imaging scenes, and the simulation results are shown in Figure 6.

According to Figure 6, the azimuth phase error curves are directly shown as the characteristics of the sine function, and the maximum error is less than π/4. Thus, the imaging precision in azimuth can be well satisfied with the approximate polynomial fitting of the slant range.

### 4.2. ETF Imaging Algorithm

Based on the derived optimal slant range model of the hypersonic platform SAR, the ETF imaging algorithm is further designed to process the echo signal of the equivalent single channel SAR system.

Assume that DPCAM SAR for hypersonic platform transmits the chirp signal. Consequently, the two-dimensional baseband signal can be given as

(21) $$\begin{array}{ll} s_{arc}(\tau ,\eta ) & =W_{1}\exp\left(j\pi K_{r}{\left(\tau -2R_{a}(\eta )/c\right)}^{2}\right){\tilde{h}}_{a}\left(\eta \right)\\ & =W_{1}\exp\left(j\pi K_{r}{\left(\tau -2R_{a}(\eta )/c\right)}^{2}\right)\exp\left(-j\pi 4\pi R_{a}(\eta )/\lambda \right) \end{array},$$

where $W_{1}=\sigma_{r}w_{r}(\tau -2R_{a}(\eta )/c)w_{a}(\eta -\eta_{c})$, and $\sigma_{r}$ is the backscatter coefficient of the targets, λ is the wavelength, c denotes the speed of light, $K_{r}$ represents the range frequency rate, τ accounts for the range time, and $w_{r}$ and $w_{a}$ are the envelope of the range and azimuth, respectively. 

The range Fourier transform of Equation (21) is performed using principle of stationary phase (POSP); then, the two-dimensional signal in range frequency domain and azimuth time domain is given by

(22) $$S_{arc}(f_{\tau },\eta )=W_{2}\exp\left(-j4\pi \left(f_{c}+f_{\tau }\right)R_{a}\left(\eta \right)/c\right)\exp\left(-j\pi f_{\tau }^{2}/K_{r}\right),$$

where $W_{2}=\sigma_{r}w_{r}(f_{\tau }/K_{r})w_{a}(\eta -\eta_{c})$, $f_{\tau }$ and $f_{c}$ represent the range frequency and carrier frequency, respectively. 

Furthermore, a closed form solution of the azimuth Fourier transform of Equation (22) is derived using POSP again. Here, the azimuth Fourier transform is given by

(23) $$\begin{array}{ll} S_{arc}(f_{\tau },f_{\eta }) & =\int_{-\infty }^{\infty }S_{arc}\left(f_{\tau },\eta \right)\exp\left(-j2\pi f_{\eta }\eta \right)d\eta \\ & =\int_{-\infty }^{\infty }\exp\left(-j\pi \left(4\left(f_{c}+f_{\tau }\right)R_{a}\left(\theta \right)/c+2f_{\eta }\theta /\omega_{s}+f_{\tau }^{2}/K_{r}\right)\right)d\theta \end{array},$$

where $f_{\eta }$ is the azimuth frequency.

From Equation (23), the phase of the Fourier integrand is

(24) $$\Theta =-\frac{4\pi (f_{c}+f_{\tau })R_{a}\left(\theta \right)}{c}-2\pi f_{\eta }\frac{\theta }{\omega_{s}}-\frac{\pi f_{\tau }^{2}}{K_{r}}.$$

To further simplify the calculation, the range variable is set as $R_{s}=\sqrt{L^{2}+r^{2}-2Lr\beta_{0}+h^{2}}$; then, Equation (18) is rewritten as

(25) $$R_{a}\left(\theta \right)=\sqrt{R_{s}^{2}-2Lr\beta_{1}\theta^{2}}.$$

Substituting Equation (25) into Equation (24), we can obtain the derivative of Θ with respect to θ, i.e.,

(26) $$\frac{d\Theta }{d\theta }=\frac{8\pi (f_{c}+f_{\tau })Lr\beta_{1}\theta }{c\sqrt{R_{s}^{2}-2Lr\beta_{1}\theta^{2}}}-2\pi \frac{f_{\eta }}{\omega_{s}}.$$

When dΘ/dθ=0, the stationary phase angle is

(27) $$\theta^{*}=\frac{cf_{\eta }R_{s}}{4\left(f_{c}+f_{\tau }\right)Lr\beta_{1}\omega_{s}\sqrt{1+c^{2}f_{\eta }^{2}/\left(8{\left(f_{c}+f_{\tau }\right)}^{2}Lr\beta_{1}\omega_{s}^{2}\right)}}.$$

Substituting Equation (27) into Equation (23), we can get the approximate two-dimensional spectrum of DPCAM SAR for hypersonic platform as follows:

(28) $$S_{arc}(f_{\tau },f_{\eta })=W_{3}\exp\left(-j\left(\frac{4\pi R_{s}\left(f_{c}+f_{\tau }\right)}{c}\sqrt{1+\frac{c^{2}f_{\eta }^{2}}{8{\left(f_{c}+f_{\tau }\right)}^{2}Lr\beta_{1}\omega_{s}^{2}}}+\frac{\pi f_{\tau }^{2}}{K_{r}}\right)\right),$$

where $W_{3}=A\sigma_{r}W_{r}(f_{\tau })W_{a}(f_{\eta }-f_{\eta_{c}})$; here, $W_{a}(f_{\eta }-f_{\eta_{c}})$ denotes the envelope of the azimuth spectrum, which is centered at the Doppler centroid frequency $f_{\eta_{c}}$.

Based on the spectrum expression of SAR baseband signal, assume that the center of the imaging scene is set as the reference range $R_{ref}$; then, the exact transfer function of the ETF imaging algorithm is developed as

(29) $$H_{etf}^{*}\left(f_{\eta },f_{\tau };R_{ref}\right)=\exp\left(j\pi \left(\frac{4R_{ref}\left(f_{c}+f_{\tau }\right)}{c}\sqrt{1+\frac{c^{2}f_{\eta }^{2}}{8{\left(f_{c}+f_{\tau }\right)}^{2}Lr\beta_{1}\omega_{s}^{2}}}+\frac{f_{\tau }^{2}}{K_{r}}\right)\right).$$

The exact transfer function multiplication has the effect of canceling the phase at the reference range, which can achieve the full focus of the targets in the range. However, since SAR echo data is dependent on the variability of the range, there exists azimuth residual phase at the non-reference range, then the azimuth image will have a different degree of defocusing. Thus, in the Doppler domain, the residual phase is further compensated to fulfill the focusing of the targets at the non-reference range. The compensation transfer function is designed as

(30) $$H_{\Delta \Phi }\left(f_{\eta },R_{s}\right)=\exp\left(j\pi \left(\frac{4\left(R_{s}-R_{ref}\right)}{\lambda }\sqrt{1+\frac{c^{2}f_{\eta }^{2}}{8Lr\beta_{1}\omega_{s}^{2}f_{c}^{2}}}\right)\right).$$

After the processing of the compensation function multiplication and inverse Fourier transform in azimuth, the focused image of targets at the non-reference range can be finally realized.

To achieve the full focus of targets near swath edge at the non-reference range, it is worth noting that the ETF imaging algorithm generally constrains the maximum non-reference range relative to the center of the SAR scene. That is, the difference of the maximum range cell migration at the non-reference range and the reference range should be less than the range resolution, meaning that

(31) $$\left|\sqrt{{\left(R_{ref}+\Delta R\right)}^{2}-2Lr\beta_{1}\theta^{2}}-\sqrt{R_{ref}^{2}-2Lr\beta_{1}\theta^{2}}-\Delta R\right|<\rho_{r}=\frac{c}{2B_{r}},$$

where $\rho_{r}$ denotes the range resolution, and $B_{r}$ represents the bandwidth of the transmit signal. 

Here, to further satisfy the requirement of SAR wide-swath imaging for hypersonic platform, the range swath can be divided into several sub-swaths based on Equation (31), and the center of each sub-swath is set as the reference range. 

**Figure 7:**
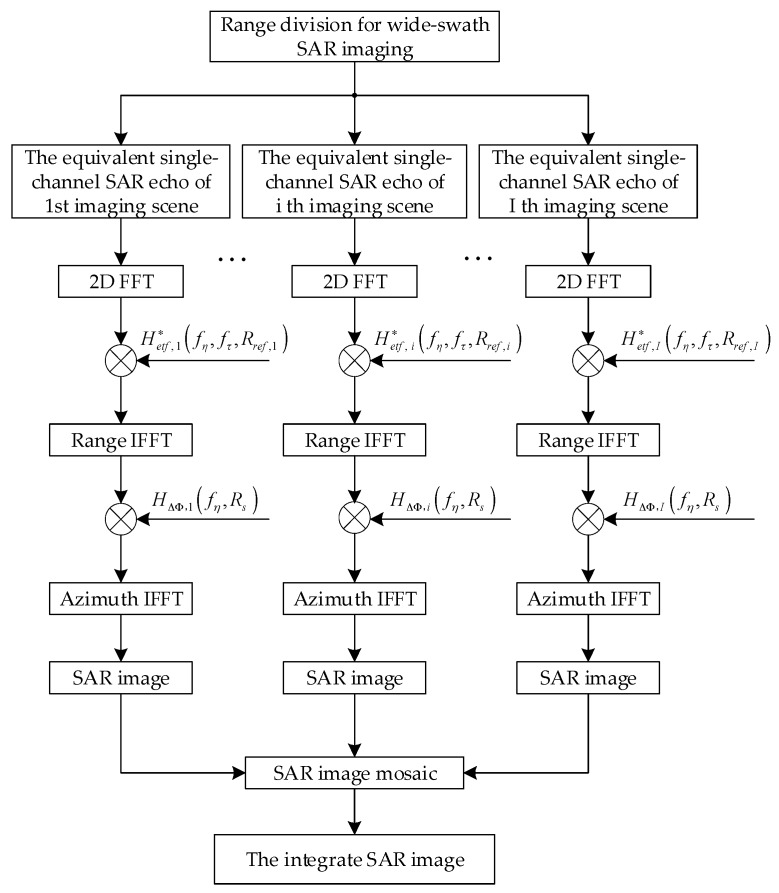
The flowchart of the ETF imaging algorithm for hypersonic platform SAR.

Based on the analysis above, the major operations of the ETF algorithm for HRWS SAR imaging are shown in Figure 7, where $H_{etf,i}^{*}(f_{\eta },f_{\tau };R_{ref,i})$ and $H_{\Delta \Phi ,i}(f_{\eta },R_{s})$ represent the exact transfer function and the compensation transfer function of the ith imaging scene, respectively.

## 5. Simulation Results and Analysis

**Figure 8:**
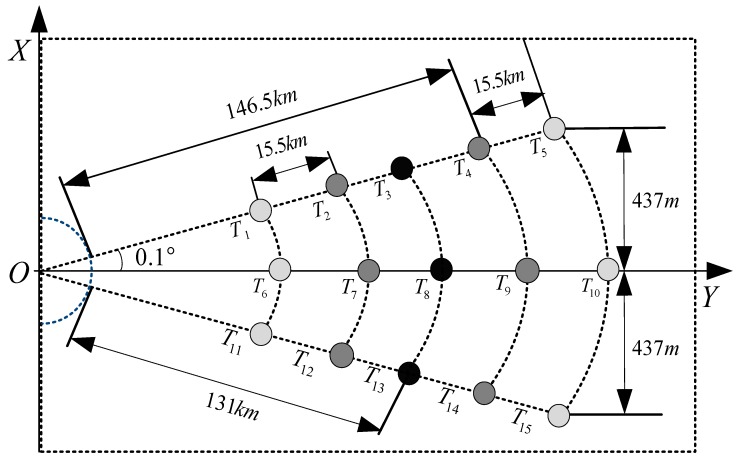
Point targets distribution for SAR imaging simulation.

In this section, we simulate a squint side-looking SAR with the stripmap imaging mode for the hypersonic platform, where the simulation parameters are listed in Table 1. Specifically, the DPCAM azimuth spectrum reconstruction algorithm is first implemented, and the azimuth pulse response of the reconstructed result is compared with the result of the single channel sampling. Furthermore, as shown in Figure 8, several point targets at different locations are distributed according to the platform characteristics and the wide-swath imaging requirement; then, the imaging algorithm is implemented. Finally, the imaging quality of the targets is analyzed to verify the effectiveness of the ETF algorithm.

As shown in Figure 8, to achieve a wide swath of 70 km on the ground, 15 point targets are distributed in the SAR imaging scene, including the far and the near edge regions. Here, the illuminated central targets are marked as the dark circle points, and the chroma of the edge targets are weakest. Meanwhile, according to the constraints of the ETF imaging algorithm and the SAR system simulation parameters, the wide swath of 70 km is divided into three imaging scenes, each of which will be overlapped.

### 5.1. Spectrum Reconstruction Results

**Figure 9:**
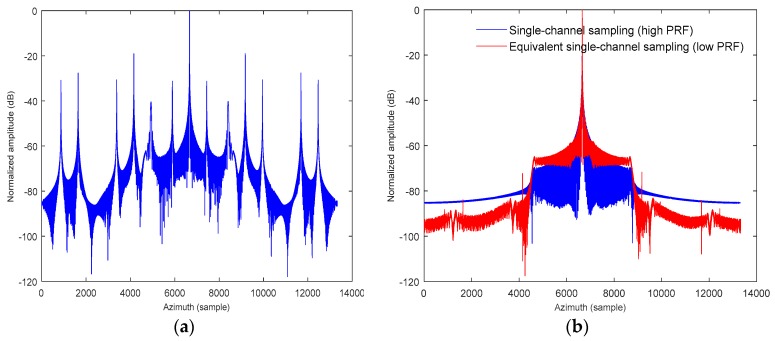
The azimuth response of DPCAM SAR for hypersonic platform: (**a**) without spectrum reconstruction; (**b**) with spectrum reconstruction.

The result of the azimuth spectrum reconstruction is shown in Figure 9. From Figure 9a, the under-sampled azimuth echo signal will result in serious spectrum ambiguity without the azimuth multichannel spectrum reconstruction processing. Then, pairs of ghost objects occur on both sides of the targets after the azimuth pulse compression, which seriously affects the imaging quality of the targets.

The proposed spectrum reconstruction algorithm is used to restore the uniform azimuth signal. The azimuth pulse response of the reconstructed result is shown in Figure 9b. It can be observed that the ambiguous energy from pairs of ghost objects is effectively suppressed, and the normalized amplitude is close to −65 dB relative to the true point targets. Meanwhile, the azimuth response of single channel sampling with a high PRF is compared with the equivalent single channel sampling with a low PRF after the spectrum reconstruction, where the target signal is well retained, and the sidelobe signal of the false targets is effectively suppressed for the equivalent single channel SAR system.

### 5.2. Spectrum Reconstruction Results

**Figure 10:**
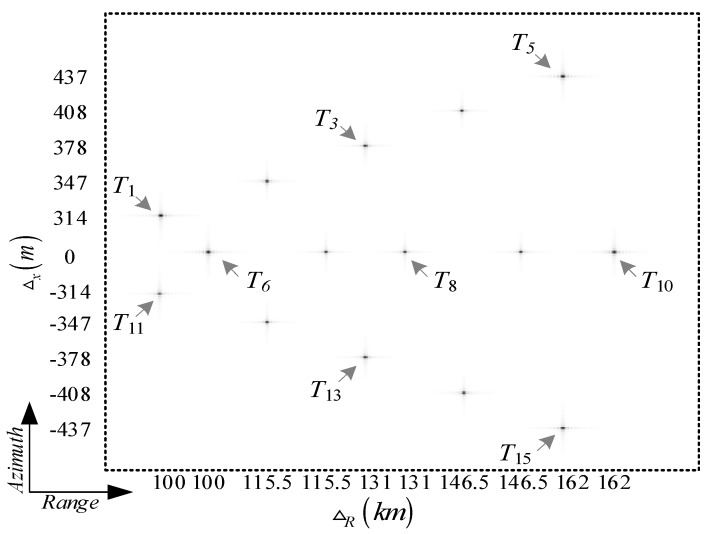
Point targets imaging results for hypersonic platform SAR.

Combining the SAR system parameters setting and the locations of targets in the imaging scene, the ETF imaging algorithm is further studied. The imaging results of multi-point targets are shown in Figure 10.

**Figure 11:**
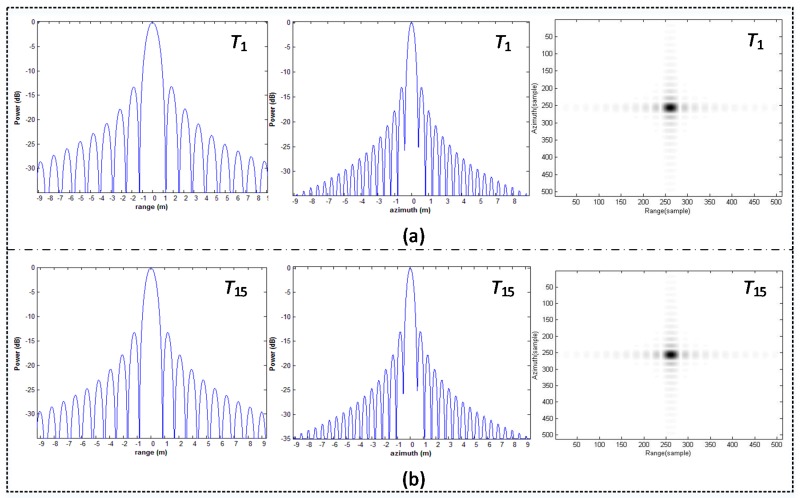
Range and azimuth response of targets near swath edge: (**a**) nearest target; (**b**) farthest target.

As shown in Figure 10, the integrate SAR image is obtained after the ETF image processing for each sub-swath and the subsequent images mosaic processing. Meanwhile, the multi-point targets located in a wide swath are well focused. Here, consider that the point targets located at the center of the imaging scene can be precisely focused, thus the imaging results of the point targets near swath edge are further analyzed. As shown in Figure 11, the range and the azimuth response of the point targets $T_{1}$ and $T_{15}$ are given in detail, respectively.

Moreover, as shown in Table 2, the performance parameters such as the impulse response width (IRW), peak sidelobe ratio (PSLR) and the integrated sidelobe ratio (ISLR) are calculated to estimate the imaging quality of the multi-point targets, which represents the different locations in the SAR scene. Meanwhile, according to Equation (7) and the parameter of the signal bandwidth listed in Table 1, the ideal IRWs of the hypersonic platform SAR are 0.886 m in range and 0.66494 m in azimuth, respectively.

As shown in Table 2, when the ETF imaging algorithm is utilized to process the SAR echo signal of the hypersonic platform with curved trajectory, the imaging quality of the point targets is close to the theoretical value in range. Meanwhile, the point targets approaching the scene center derive a high imaging performance, while the corresponding quality of the point targets near swath edge is slightly lower, but it can basically meet the requirement of azimuth resolution.

### 5.3. Simulation Results for Complex Scenes

**Figure 12:**
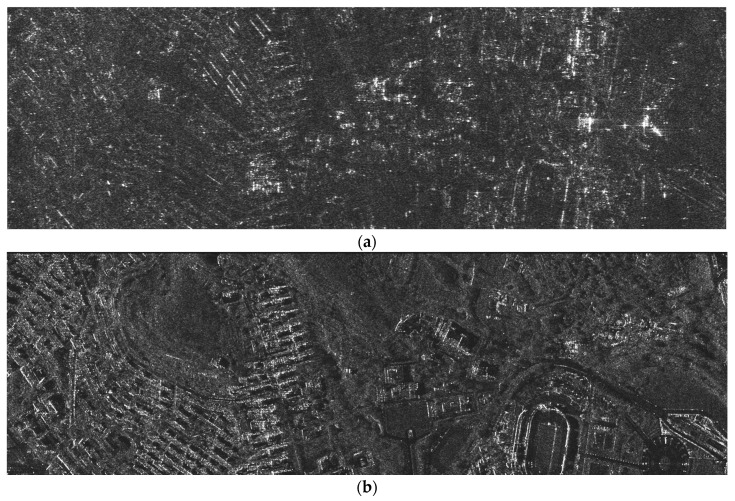
Simulation results for complex scenes using TerraSAR-X real data: (**a**) imaging result processed with the echo data of the first sub-aperture; (**b**) imaging result of the proposed method.

To further verify the effectiveness of the proposed method, the imaging processing simulation for complex scenes is carried out using real TerraSAR-X image data. Here, the resolution of TerraSAR-X image data is 1 m and the scene is 5 km (azimuth) × 10 km (range). Specifically, the complex image data are used as the scattering coefficients of scenes, and then the multichannel SAR echo data of the hypersonic platform can be simulated according to the imaging geometry shown in Figure 1 and the simulation parameters listed in Table 1. Furthermore, the imaging processing is performed using the proposed ETF algorithm, which is shown in Figure 7. Finally, the imaging results of partial scenes are shown in Figure 12b. Meanwhile, as a comparison, the imaging result obtained by the first sub-aperture is also provided in Figure 12a. It can be clearly seen that the defocus and azimuth ambiguity are obvious in SAR images because the PRF is lower than the Doppler bandwidth of echo signal. However, in Figure 12b, we can see that the azimuth ambiguity is effectively suppressed using the proposed multichannel HRWS imaging algorithm, and that the SAR image for complex scenes is well focused.

## 6. Conclusions

Combined with the motion characteristics of the hypersonic platform SAR, this paper establishes the imaging geometry model matched with the curved trajectory of this platform, and designs the azimuth multichannel sampling model based on the DPCA technique. When the imaging algorithm is explored to process the SAR echo signal, the high-efficiency spectrum reconstruction method based on the discrete Fourier transform is executed to pre-eliminate the ambiguous spectrum. Then, the uniform azimuth data of the equivalent single channel SAR system is developed. Furthermore, the approximate slant range model is derived to reduce the imaging complexity based on the minimax criterion, and the optimal second-order approximate coefficients of the cosine function are obtained using the two-population coevolutionary algorithm. Simulation results show that the approximate processing of the SAR slant range well satisfies the imaging accuracy in azimuth. On this basis, the ETF imaging algorithm is designed for a hypersonic platform SAR with curved trajectory, and the method of range division is further used to achieve the HRWS imaging. Simulation results of point targets validate the effectiveness of the ETF imaging algorithm.

## Figures and Tables

**Table 1:** Simulation parameters setting of SAR system.

Parameters	Values
Platform velocity	6 Ma
Platform height	60 km
Turning radius	100 km
Center of slant range	131 km
Frequency	10 GHz
Signal bandwidth	150 MHz
Pulse width	10 us
Oversampling factor (azimuth)	1.2
Oversampling factor (range)	1.4
Beamwidth (sub-aperture)	2.8648°
Length of sub-aperture	0.6 m
Number of sub-apertures	7
Doppler Bandwidth	6800 Hz
Ideal PRF	1165.7 Hz
System PRF	1398.9 Hz

**Table 2:** Imaging performance analysis for hypersonic platform SAR.

Targets	IRW (m) Range/Azimuth	PLSR (dB) Range/Azimuth	ISLR (dB) Range/Azimuth
*T*_1_	0.883/0.67	−13.22/−13.17	−9.81/−9.10
*T*_3_	0.883/0.66	−13.24/−13.21	−9.82/−9.12
*T*_5_	0.884/0.69	−13.19/−13.17	−9.88/−8.78
*T*_6_	0.884/0.69	−13.18/−13.13	−9.83/−9.03
*T*_8_	0.882/0.66	−13.25/−13.24	−9.80/−9.17
*T*_10_	0.884/0.68	−13.22/−13.18	−9.87/−9.07
*T*_11_	0.883/0.68	−13.20/−13.18	−9.79/−9.11
*T*_13_	0.882/0.67	−13.25/−13.23	−9.81/−9.11
*T*_15_	0.883/0.68	−13.20/−13.16	−9.87/−8.98
